# Cyclic Stretch of Either PNS or CNS Located Nerves Can Stimulate Neurite Outgrowth

**DOI:** 10.3390/cells10010032

**Published:** 2020-12-28

**Authors:** Vasileios Kampanis, Bahardokht Tolou-Dabbaghian, Luming Zhou, Wolfgang Roth, Radhika Puttagunta

**Affiliations:** 1 Laboratory for Experimental Neuroregeneration, Spinal Cord Injury Center, Heidelberg University Hospital, 69118 Heidelberg, Germany; Vasileios.Kampanis@med.uni-heidelberg.de (V.K.); Bahardokht.TolouDabbaghian@med.uni-heidelberg.de (B.T.-D.); 2 Laboratory of NeuroRegeneration and Repair, Hertie Institute for Clinical Brain Research, University of Tübingen, 72076 Tübingen, Germany; lumingzhou86@gmail.com; 3 Laboratory for Experimental Neurorehabilitation, Heidelberg University Hospital, 69118 Heidelberg, Germany; Wolfgang.Roth@med.uni-heidelberg.de

**Keywords:** mechanical loading, cyclic stretch, PNS, CNS, neurite outgrowth, DRG

## Abstract

The central nervous system (CNS) does not recover from traumatic axonal injury, but the peripheral nervous system (PNS) does. We hypothesize that this fundamental difference in regenerative capacity may be based upon the absence of stimulatory mechanical forces in the CNS due to the protective rigidity of the vertebral column and skull. We developed a bioreactor to apply low-strain cyclic axonal stretch to adult rat dorsal root ganglia (DRG) connected to either the peripheral or central nerves in an explant model for inducing axonal growth. In response, larger diameter DRG neurons, mechanoreceptors and proprioceptors showed enhanced neurite outgrowth as well as increased Activating Transcription Factor 3 (ATF3).

## 1. Introduction

During the development of multicellular organisms, tissues form and grow under the presence of mechanical forces [1,2]. The cytoskeleton receives and responds to mechanical cues of neighboring cells, the extracellular matrix or exogenous factors [2,3]. The nervous system is subjected to mechanical forces that guide its development, form its shape and act as cues for the perception of the internal physiology of the body (proprioception) or the outside world (mechano-sensation) [1,4,5]. During development, growing axons of nascent neurons reach their targets prior to the completion of the overall growth of the body [6], however, their neurites continue elongating during this growth period through the application of tensile strain [7]. The mature central nervous system (CNS) is enclosed in a stiff bony structure, either the skull or the vertebral column, providing protection as well as shape. In contrast, the mature peripheral nervous system (PNS) is not enveloped by bone but rather intermingled with internal organs and tissues, therefore more susceptible to mechanical cues. In addition to the morphological differences between the two nervous systems, it is known that the CNS of mammals has a limited regenerative capacity leading to permanent functional loss after a severe injury. At the same time, the PNS retains its ability to re-grow functional connections after a lesion.

Interestingly, in vitro studies have shown that cultured neurons achieve long-distance elongation when subjected to tensile forces [8,9,10]. Pfister et al. have shown that embryonic dorsal root ganglia (DRG) neurons in culture can reach extreme levels of elongation, up to 5 cm when their axons are progressively and repetitively stretched for several days (1 mm the first day was the net result of 2 µm displacements every 172 s which was altered each successive day up until 14 days), similar to towing forces observed during development [11]. Likewise, adult DRG neurons retain the capacity to respond to tensile forces as they can reach profound levels of elongation when stretched in vitro [10]. This validates that adult neurons retain the capacity to respond to tensile forces even after maturity.

The movement of limbs subjects respective nerves to a cyclic mechanical stretch of a limited amplitude [12], although the precise nerve lengthening in vivo by limb movement is poorly reviewed. Post-mortem studies on cadavers have shown that different nerves are stretched by the movement of the limbs they are innervating, with stretch ranging from 0 to 15% [13,14,15,16,17,18,19,20,21,22]. Therefore, given the initial responsiveness of growing neurons to mechanical stretch and the absence of regenerative capacity of the adult CNS, we questioned whether the lack of mechanical cues plays a role in the failure of the CNS to regenerate upon injury.

In the aforementioned in vitro studies, neurons were stretched by towing of growth cones and elongation of neurites as the extension distance grew, similar to that seen during development. However, this does not recapitulate the physiological conditions of normal limb movement, where the nerves are stretched cyclically by the extension and contraction of the respective muscles, without towing extended distances. With SH-SY5Y human neuroblastoma cells, S. Higgins et al. showed that 10% cyclic mechanical stretch was enough to produce statistically significant longer neurites than neurons that were not stretched [23]. Although, closer to normal stretch conditions indicated in vivo, their approach is far from similar to the conditions in vivo as the cultures consist of dissociated cells. Nevertheless, they showed that when cells are let to grow for two days on a collagen-coated membrane before 10% equiaxial stretching at 0.25 Hz for 120 min/d for seven days, it led to increased neurite outgrowth similar to the level induced by the neurogenic factor, retinoic acid (10 µM).

Interested in producing a more physiological stretch seen from muscle tension on nerves rather than a mechanical strain on whole neurons, including the soma, as observed in the in vitro work, our study developed a novel explant nerve-DRG model. With this model, we wanted to address whether the mechanical flexibility of the PNS contributes to its regenerative potential and if, applied onto CNS located nerves, if it can enhance the outgrowth of adult sensory neurons after stretch. To compare the differences between the peripheral and centrally located nerves responding to the tensile forces, we used the lumbar DRG connected to either their peripheral nerve (the sciatic nerve) or their central nerve branches. Using a custom-built bioreactor, we found a specific stretch amount that did not lead to further nerve damage but increased overall outgrowth in our CNS located nerve-DRG model. Moreover, this was further pronounced in large diameter neurons also from the PNS nerve-DRG model and was not observed from the nociceptive subpopulation. We confirm that either mechanoreceptors or proprioceptors or both respond to mechanical stimulation. Mechanistically we found the Activating Transcription Factor 3 (ATF3) to be upregulated by the outgrowth-inducing stretch-driven signal.

## 2. Materials and Methods

### 2.1. Animals

For this study, adult female or male Fischer-344 rats (8–12 weeks old, Janvier Labs, Le Genest-Saint-Isle, France) were used, and all animals were maintained daily under controlled temperature (22 ± 1 °C) and humidity (45–65%) conditions. All animals were sacrificed, and experiments were conducted in accordance with the European Union Directive (2010/63/EU) and institutional guidelines. Animals were sacrificed by intraperitoneal injection of an overdose of a mixture of ketamine (5.2% *v*/*v*; Bremer Pharma, Warburg, Germany) and xylazine (13.2% *v*/*v*; CP-Pharma, Burgdorf, Germany), in 0.9% *v*/*w* NaCl followed by decapitation.

### 2.2. Sciatic Nerve and Central Nerves Connected to L4-L5 DRG Explants

Upon decapitation, the skin and viscera were removed, sparing the spinal column and the hind limbs. The cadaver was fixed in the supine position and the lumbosacral plexus nerves were exposed ventrally by removal of the surrounding muscles and connective tissue. The sciatic nerve was identified. The ventral half of the spinal vertebral column was carefully removed using Rongeurs forceps (FST, 16021-14, Foster City, CA, USA) without damaging the spinal cord or the DRG. For the bifurcating sciatic nerve to L4-L5 DRG explant, the nerve was cut 2.5 cm distal to the DRG in the periphery and their central nerves were cut in proximity to the DRG. For the central nerves connected to either the L4 or L5 DRG explant, the nerves were cut 2.5 cm rostral to their respective DRG and their peripheral connection was cut in proximity to the DRG. The explants were kept in ice-cold Mg^2+^- and Ca^2+^-free Hank’s balanced salt solution (HBSS, Merck KGaA, L2045, Darmstadt, Germany). Each DRG-nerve explant was mounted on two poly-ether-ether-ketone (PEEK, 11 mm × 4 mm × 4 mm, Zentralbereich INF367, University of Heidelberg, Heidelberg, Germany) bars fixed in the bioreactor with screws. The two opposing PEEK bars were placed 1 cm apart and the explants were glued on each bar using an acrylic adhesive (CYANO-Fast, 5 g, Hagen-Werken, 152261, Duisburg, Germany), generally used for oral work. The explant was kept in a metal container (7.5 cm × 2.5 cm × 2.0 cm) filled with a solution of F12/DMEM (Thermo Fischer Scientific, 31330038, Waltham, MA, USA), 10% fetal bovine serum (FBS, Merck KGaA, F9665, Darmstadt, Germany), 1× B27 serum-free supplement (Thermo Fischer Scientific, 17504044, Waltham, MA, USA), 1% Pen/strep (Sigma-Aldrich, P4333, Waltham, MA, USA) and was placed into a mechanical stretch modified incubator (Invivo2 400 Hypoxia incubator, I&L Biosystems GmbH, Königswinter, Germany) for 3 h at 37 °C, 5% CO_2_. The explants for the static control group were kept in the same solution and incubation conditions. For consistency, control, and stretch group DRG were dissected and incubated the same day. Due to the lack of a second bioreactor, the DRG in the non-stretch group were not attached to a static bioreactor but instead were glued to a Petri dish 60.mm Ø (Orange Scientific, 5550200, Braine-l’Alleud, Belgium) without any mechanical tension applied and kept in the same incubator for the same time as the stretched group.

### 2.3. Tensile Stretch Incubator

**Figure 1:**
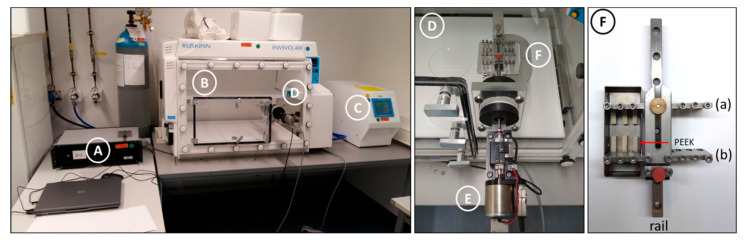
In-house built setup for the mechanical stretch of nerve explants. (**A**) The controller is connected to GalilTools software and the oscillator (**D**,**E**). (**B**) The incubator chamber keeping constant culture conditions. (**C**) The gas mixer analyzing the air constitution of the incubator chamber, 5% CO_2_. (**D**) The bioreactor (**F**) is mounted on the oscillator (**E**). (**E**) The oscillator is connected to the controller (**A**) and the bioreactor (**F**) inside the incubator. (**F**) Bioreactor. Two metal plates (**a** and **b**) are built on a metal rail, and poly-ether-ether-ketone (PEEK) bars are fixed on them by screws and kept in a metal boat filled with culture medium. Metal plate b is connected to the oscillator, moving back and forth, while the other plate is fixed to the stage and the metal rail.

For the mechanical stretch of the DRG-nerve explants, a bioreactor was built in-house. As shown in Figure 1F, the metal plate (a) (8 cm × 4 cm × 2.5 cm) was fixed on a metal rail (16 cm). Another metal plate, (b), with the same dimensions as (a), was also built on the same rail, free to move back and forth to change the distance from plate (a) but able to be fixed on the rail by a screw to keep the distance from plate (a) constant when needed. Each plate had small square openings (4 mm × 4 mm × 1 cm) next to each other to fit the PEEK bars, which could be fixed to the plates by small screws. The ends of the PEEK bars were facing towards each other. The bars could maintain a distance of 1 cm apart by adjusting plate (b). After nerve explants were glued on the fixed PEEK bars, the bioreactor was fixed to the oscillator (Renishaw, Gloucestershire, UK) which could move the plate (b) back and forth at a specific amplitude and frequency, able to move away or towards the fixed plate (a). Half of the oscillator resided inside the incubator chamber, controlling the movement of the plate (b) and the other half sat outside the incubator’s plexiglass door connected to an electronic controller (August Steinmeyer GmbH & Co. KG, Albstadt, Germany). The controller was connected to a laptop running the GalilTools program (Galil Motion Control Inc., Rocklin, CA, USA), which was used to set the parameters of the cyclic movement of the oscillator/bioreactor. The nerves were cyclically stretched with either 10% or 20% mechanical strain. 1 cm separated the two glued spots, thereby, 10% stretch was achieved with 1 mm dislocation towards the long axis of the nerve and 20% stretch with 2 mm dislocation, respectively.

### 2.4. GalilTools Programming Code Used for Oscillatory Movement

The cyclic movement of the oscillator was controlled by the GalilTools program and the code used is shown in the supplementary information (Supplementary File S1). This program controls the movement of the oscillator with an amplitude radius of 1 mm and a frequency of 0.5 Hz. Modifying the VS and the radius, we could achieve different amplitudes and frequencies.

### 2.5. DRG Cultures

Following 3 h of a cyclic stretch of the DRG-nerve explants, the DRG were dissected and kept in ice-cold Mg^2+^- and Ca^2+^-free HBSS. DRG were spun down at 300× *g* for 1 min. HBSS was replaced by 500 μL of a digestion mixture of 2.5 mg/mL collagenase (Merck KGaA, C9407, Darmstadt, Germany) and 5 mg/mL neutral protease (dispase) (Worthington Biochemical Corp., LS02104, Lakewood, NJ, USA) in HBSS and incubated at 37 °C for 35 min by gently shaking every 10 min. Then, the DRG were centrifuged at 300× *g* for 2 min, and the digestion solution was discarded. The DRG were washed once with prewarmed DRG medium consisting of DMEM/F12, 10% FBS, and 1× B27 serum-free supplement solution. DRG were centrifuged at 300× *g* for 2 min, the medium was discarded and fresh DRG medium was added to mechanically triturate them by a fire-polished glass pipette to a single cell solution. DRG cells were centrifuged at 300× *g* for 5 min, the supernatant was discarded, and the cells were resuspended in DRG medium. 100 μL of the single-cell resuspension was loaded onto a 15 mm-diameter glass coverslip (VWR, 631-1579, Radnor, PA, USA), coated with 100 μg/mL poly-L-ornithine (PLO, Merck KGaA, P3655, Darmstadt, Germany) and 2 μg/mL laminin (Merck KGaA, L2020, Darmstadt, Germany), at a density of 4000 cells/coverslip. DRG cells were incubated at 37 °C 5% CO_2_ for 1 h, then each well was slowly and carefully brought up to 1000 μL with DRG culture medium consisting of DMEM/F12, 1× B27 supplement, and 1% Pen/Strep. Cells were incubated for 17 h and fixed for 20 min with ice-cold 4% PFA (Carl Roth GmbH & Co. KG, 0355.3, Karlsruhe, Germany)/0.1 M phosphate buffer. PFA solution was discarded, cells were rinsed once with 1× TBS and kept at +4 °C in 1× TBS.

### 2.6. Immunocytochemistry

DRG neurons were permeabilized and blocked with 1% donkey serum (GeneTex, GTX73245, Irvine, CA, USA) in TBS/0.1% TritonX-100 (Tx100, neoLab Migge GmbH, Heidelberg, Germany) for 30 min at room temperature (RT). Then, cells were incubated with the primary antibodies diluted in blocking solution to the desired concentrations overnight (o/n) at +4 °C. Cells were rinsed three times for 5 min each with 1× TBS and then incubated with corresponding secondary antibodies diluted in the blocking solution for 1 h at RT. Cells were rinsed three times for 5 min each with 1× TBS and the coverslips were mounted on glass slides with Fluoromount-G (Southern Biotechnology Associates, 0100-01, Birmingham, AL, USA). Primary antibodies used for this study: β-III-tubulin (1:2000, mouse, Promega GmbH, G7121, Walldorf, Germany), CGRP (calcitonin gene-related peptide, 1:200, rabbit, Immunostar, 24112, Hudson, WI, USA), TrkC (tropomyocin receptor kinase C, 1:200, goat, R&D systems, AF1404, Minneapolis, MN, USA), Neurofilament 200 (1:1000, rabbit, Abcam, Red No. ab8135, Cambridge, UK), Parvalbumin (1:300, rabbit, Swant, PV27, Marly, Switzerland), TrkB (tropomyocin receptor kinase B, 1:250, goat, R&D systems, AF1494, Minneapolis, MN, USA) Secondary antibodies used for this study: AlexaFluor-594 (1:1000, donkey anti-goat, Life Technologies, A11058, Carlsbad, CA, USA), AlexaFluor-488 (1:1000, donkey anti-rabbit, Life Technologies, A21206, Carlsbad, CA, USA), Cy5 (1:500, donkey anti-mouse, Jackson Immuno-Research Laboratories, 715-175-151, West Grove, PA, USA). All cells were counterstained with 4,6-diminido-2-phenylindole (DAPI, 1:2000, Sigma-Aldrich, MBD0015, St. Louis, MO, USA).

### 2.7. Immunohistochemistry

Lumbar DRG-sciatic nerve/central branch explants were dissected as described above (see Section 2.5). Following 3 h of 10% mechanical stretch, DRG were dissected from their respective nerves and fixed with 4% PFA for 4 h at RT. Then, DRG were washed once with 1× PBS and then kept in 30% sucrose solution for 24 h at +4 °C. DRG were cryosected in 18 μm-thick sections. Sections were permeabilized in 0.25% TritonX-100/1× TBS solution and blocked in 5% donkey serum/1× TBS solution for 2 h at RT. Sections were incubated o/n with the primary antibody, anti-ATF3 (Activating Transcription Factor 3, 1:200, rabbit, Novus Biologicals, NBP1-85816, Littleton, CO, USA), anti-H3K9K14ac (Histone 3 lysine 9 lysine 14 acetylation, 1:1000, rabbit, Sigma Aldrich, ZRB06599, St. Louis, MO, USA), and β-III-tubulin (1:2000, mouse, Promega GmbH, G7121, Walldorf, Germany), diluted in 0.25% TritonX-100/1% donkey serum in 1× TBS solution. DRG were washed three times with 1× TBS and then incubated for 1 h at RT with the secondary antibody, AlexaFluor-594 (1:1000, donkey anti-rabbit, Life Technologies, A21207, Carlsbad, CA, USA), AlexaFluor-488 (1:1000, donkey anti-mouse, Life Technologies, A21202, ThermoFischer, MA, USA). DRG were washed three times with 1× TBS and counterstained with DAPI for 15 min. Sections were washed once with 1× TBS and coverslipped with Fluoromount-G.

### 2.8. Nerve Cryosectioning and Hematoxylin-Eosin (H&E) Staining

After the mechanical stretch, the 1 cm-long nerve portion between the two glued PEEK bars was collected, fixed with 4% PFA for two hours at room temperature, washed once with 1× TBS, immersed into 30% sucrose (Carl Roth GmbH & Co. KG, 9286.2, Karlsruhe, Germany)/0.1 M phosphate buffer and stored at +4 °C until sectioning. Nerves were immersed in Tissue-Tek O.C.T compound (VWR, SAKU4583, Radnor, PA, USA), cut into 10 μm-thick longitudinal sections using a cryostat (knife −20 °C, object −18 °C) (Carl Zeiss AG, Oberkochen, Germany) and mounted on positively charged SuperFrost Plus glass slides (ThermoScientific, J1800AMNZ, Waltham, MA, USA). Nerves were subjected to H&E staining to check the cytoarchitecture of the tissue after stretch. Slides were rehydrated by immersion of 5 min each in 100% propanol, 96% propanol, 70% propanol, 50% propanol, and then twice with distilled water. Slides were immersed into Hemalum acid solution (Carl Roth GmbH & Co. KG, T865.1, Karlsruhe, Germany) for 3 min and washed once for 5 min with distilled water. Slices were stained with 1% Eosin solution (Merck KGaA, 117081, Darmstadt, Germany) for 3 min and washed with distilled water until contrast was achieved. Slides were dehydrated by immersion in 96% propanol for 1 min, 100% propanol twice for 1 min, and then 4 times into Roti-Histol (Carl Roth GmbH & Co. KG, 6640.1, Karlsruhe, Germany) for 5 min each. Finally, slides were coverslipped using Neo-Mount (Merck KGaA, 109016, Darmstadt, Germany) and stored at RT until imaging.

### 2.9. Imaging and Image Analysis

Photomicrographs of DRG cultures were taken with an Olympus FluoView FV1000 confocal microscope (Olympus Deutschland GmbH, Hamburg, Germany) using the FluoView imaging program at 10× magnification. The images were analyzed by Fiji (ImageJ). For neurite length analysis, the NeuronJ plugin of Fiji was used. We manually counted distinguishable individual cells in each given experiment, on average 200–400 neurons were analyzed for each group. βIII-tubulin was used to detect and trace all neurites from each neuron. From each picture, the total neurite length and diameter of each neuron were measured. For each experimental condition, the average neurite length per neuron was calculated. For neuronal diameter analysis, diameters were grouped into 0–20 μm, 20–30 μm, 30–40 μm, and 40+ μm diameter bins and the average neurite length was calculated for each group. Percentages of DRG sorted by diameter are: 26.9 ± 6% for 0–20 μm, 57.4 ± 4% for 20–30 μm, 7.4 ± 4% for 30–40 μm and 8.3 ± 3% for 40+ μm. For the analysis of the subpopulations of DRG, TrkC+ cells represented 15 ± 5% of the total counted DRG population, CGRP+ neurons represented 70 ± 5% total counted DRG population and the NF200+ neurons represent the 43 ± 7% total counted DRG population. Photomicrographs of immunohistochemically stained DRG were taken using the FluoView FV1000 confocal laser scanning microscope (Olympus Deutschland GmbH, Hamburg, Germany) with a 20× magnification. ATF3 and H3K9K14ac nuclear intensity density analysis was done by Fiji. DRG nuclei were encircled manually at the DAPI channel and the intensity density was measured at ATF3 or H3K9K14ac channel. H&E stained nerves were imaged with an upright BX53 Olympus microscope (Olympus Deutschland GmbH, Hamburg, Germany) using the brightfield illumination at a magnification of 10×. These pictures were used to assess tissue qualitatively.

### 2.10. Statistical Analysis

A paired two-tailed Student’s *t*-test was used to compare the average neurite length of all DRG between the static control and stretched subjects. DRG ATF3 and H3K9K14ac nuclear intensity density statistical analysis were also done with a paired two-tailed Student’s *t*-test. For the neurite length analysis of the different diameter DRG, a Two-way ANOVA with post-hoc Sidak’s multiple comparison test was used to compare the average neurite length between the different diameter groups between the static control and stretched subjects. Data are presented as mean ± standard error of the mean (SEM) unless otherwise noted. Statistical analysis was done using Prism 6 software (GraphPad Software Inc., La Jolla, CA, USA), with an alpha level of 0.05 for significance. The number of independent experiments is depicted by the number of points in the respective graphs, with color-coding indicates paired experiments.

## 3. Results

### 3.1. A Stretch Bioreactor was Built In-House to Apply Cyclic Mechanical Tension to DRG-Nerve Explants

To apply mechanical tension of a specific amplitude and frequency to DRG-nerve explants a bioreactor (Figure 1F) was built in-house, as no available commercial setup fulfilling our requirements was available. An oscillator (Figure 1E) controls the bioreactor sitting partially inside the incubator (Figure 1B), which is kept at a constant temperature, humidity, and CO_2_ levels (controlled by a gas mixer, Figure 1C). The outside half of the oscillator is connected to the controller (Figure 1A). This controller converts the signal of the GalilTools software into oscillatory movements directing the oscillator.

### 3.2. Mechanical Tension Applied to DRG-Nerve Explants Can Enhance Axonal Outgrowth In vitro

**Figure 2:**
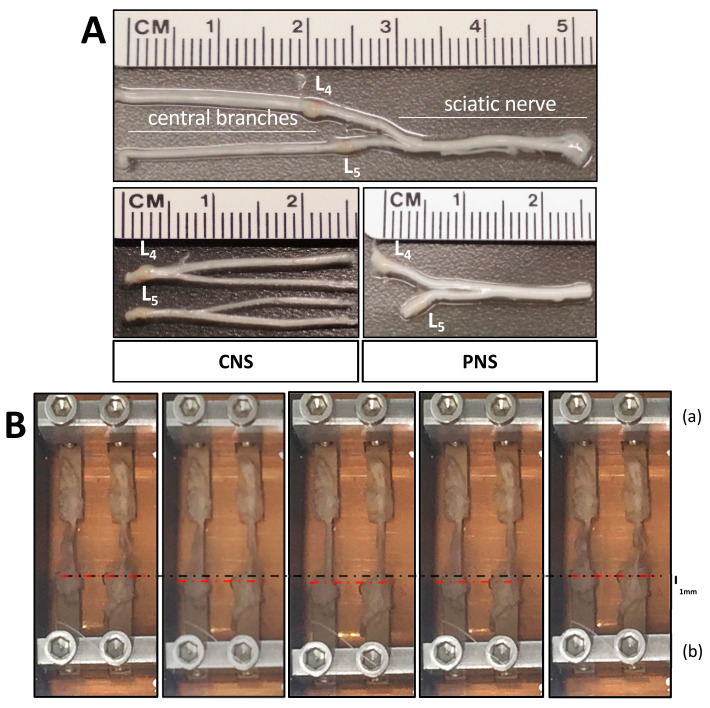
Peripheral nervous system (PNS) and central nervous system (CNS) nerve-dorsal root ganglia (DRG) explants. (**A**) The L4-L5 DRG are either connected to the sciatic nerve or continue independently along their dorsal roots into the spinal column. Two groups were prepared with either the central nerves connected to their respective L4/L5 DRG or the sciatic nerve connected to the L4/L5 DRG. (**B**) Explant nerves were glued on two PEEK bars facing towards each other, with the DRG sitting on one bar and the end of the nerve(s) seated on the other bar with a 1 cm distance between them. With the 10% cyclic movement of plate B (lower metal plate), 1 mm dislocation was achieved.

Prior to the experimentation, we needed to determine the parameters necessary to run our stretch experiments within the bioreactor. To develop our DRG-nerve explant model, we examined which part of the DRG and nerve could be contiguously dissected and withstand the strain. We began with the central side from DRG L4 and L5 of Fischer-344 rats. We could consistently extract the dorsal roots prior to entry into the spinal cord (~2.5 cm residing within the vertebral column before the dorsal root entry zone of the lumbar enlargement) without issue. An equivalent length was taken from the peripheral sciatic nerve (Figure 2A). Importantly, the part of the nerve taken between the CNS and PNS explants represents the same subpopulation of neurons. However, it is essential to note the structure of either side is not equivalent and may lead to differences observed during experimentation. We then examined how to fix the nerves to the bioreactor without additional strain directly on the DRG itself. This was accomplished using two PEEK bars and acrylic adhesive (Figure 2B). Given the diverse cell types of the explant, we next optimized the growth media (details listed within the methods) that allowed for explant viability over a period of time of mechanical strain prior to DRG culture. We observed that bioreactor use with explants longer than 3 h did not allow for survival, which limited our time for cyclic stretch and recovery time. Recovery time indicates the time after mechanical loading has ceased and prior to DRG culture. Finally, we needed to establish the percent of stretch that would induce a visible result in neurite length. The various iterations examined are listed in Table 1. The rationale was to allow for any signaling from the cell periphery to reach the soma. However, it was clear that total incubation times exceeding 3 h, including stretch and/or recovery, did not survive well irrespective of the stretch amplitude. Thus, we concluded the maximum explant culture to be 3 h stretch time without recovery time. 2.5% and 5% stretch were performed as single-pilot experiments, without significant differences observed, in contrast to the 10% stretch, which we continued as our regenerative stretch paradigm for the following experiments. It would be of interest to return to the lower levels of stretch and examine full data sets for conclusive comparisons.

**Figure 3:**
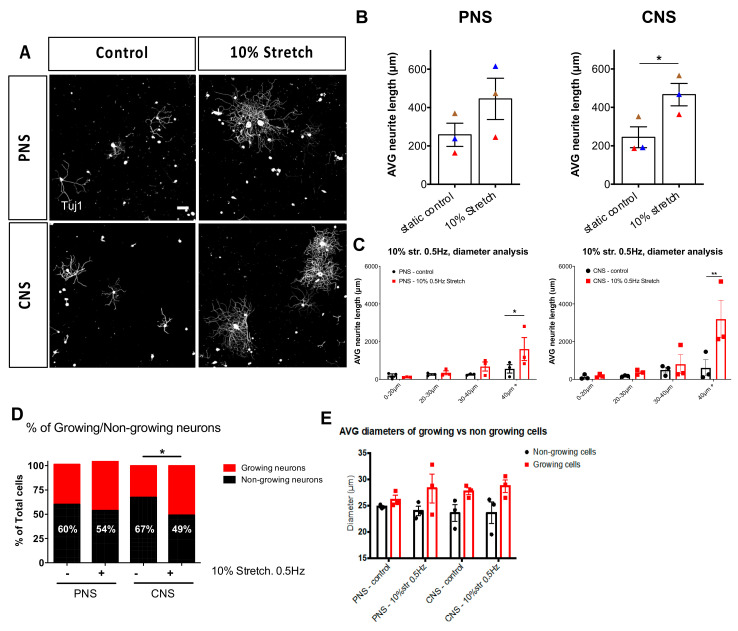
Ex vivo mechanical stretch enhances neurite outgrowth. Three hours following a mechanical stretch of either the sciatic nerve connected to L4-L5 DRG or connected to their respective central nerves, DRG were dissected and cultured for 18 h on laminin substrates. (**A**) Immunohistochemical staining of βIII-tubulin (Tuj1) was used to trace all neurites of the DRG. Scale bar, 100 μm, (**B**) 10% mechanical stretch with a frequency of 0.5 Hz enhanced neurite outgrowth of DRG derived from a CNS explant but not from a PNS explant. Paired two-tailed Student *t*-test, mean ± SEM of paired independent experiments indicated by the colors, * *p* < 0.05, (**C**) Diameter analysis of DRG outgrowth comparing the static controls to the stretched explants shows the largest neurons responding to stretch. Two-way ANOVA with post-hoc Sidak’s multiple comparison test, mean ± SEM, * *p* < 0.05, ** *p* < 0.01. (**D**) Quantification of growing vs. non-growing neurons in each group showed that non-growing neurons decreased in the CNS group upon the stretch. Two-way ANOVA with post-hoc Sidak’s multiple comparison test, * *p* < 0.05. (**E**) Examination of the average size of growing versus non-growing neurons shows non-growing neurons to be consistently smaller diameters than growing neurons.

To investigate whether mechanical strain can enhance axonal outgrowth ex vivo we subjected sciatic nerve-L4/L5 DRG or central nerves-L4/L5 DRG explants to 3 h of mechanical loading, with an amplitude of 10% (1 mm dislocation of 1 cm long nerve, Figure 2B) and a frequency of 0.5 Hz. Surprisingly, we found that mechanical stimulation of central nerves connected to their respective DRG led to enhanced neurite outgrowth of DRG (Figure 3A,B). Although seen as a trend, the sciatic nerve stretch did not significantly enhance overall neurite outgrowth (Figure 3A,B). It should be mentioned that more neurons survived in culture from DRG-sciatic nerve explants than from DRG-central nerve explants, irrespective of whether they had received stretch or not, probably due to the optimization done off the peripheral nerve-DRG explants.

The DRG is a heterogeneous population of neurons (nociceptive, mechanoreceptive, and proprioceptive neurons) that is partially sorted via diameter size. Therefore, to get an idea of which subpopulation responds to mechanical strain and should be studied further, we applied diameter analysis to the cultured DRG. We found that the larger diameter neurons (40+ μm) of the mechanically loaded groups had increased outgrowth compared to the static controls (Figure 3C). Additionally, we observed that stretch-induced the switch from non-growing neurons to growing neurons in the CNS explants (Figure 3D). Moreover, in general, those that make up the growing population of neurons represent the medium to large diameter neurons compared to the small-diameter neurons of the non-growing group (Figure 3E).

**Figure 4:**
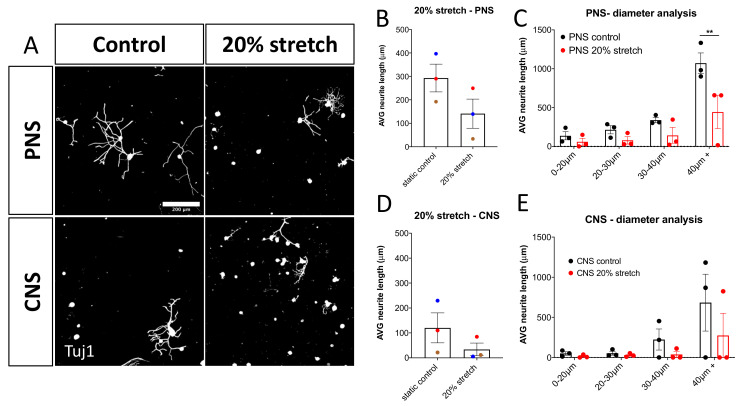
20% 0.5 Hz stretch has a negative impact on neurite outgrowth of DRG in vitro. (**A**) Representative photomicrographs (10×) of cultured rat DRG following 20% 0.5 Hz stretch or no stretch and stained for βΙΙΙ-tubulin (Tuj1). Scale bar, 200 μm. (**B**) Neurite length analysis of DRG cultured after 20% 0.5 Hz stretch of PNS-nerve explant compared to static control. Stretch appeared to have a negative impact on the neurite outgrowth, although not statistically significant. Paired two-tailed Student’s *t*-test, mean ± SEM of paired independent experiments indicated by the colors. (**C**) Diameter analysis showing a statistically significant reduction in the outgrowth of large-diameter neurons after a 20% stretch of the PNS-nerve explant. Two-way ANOVA with post-hoc Sidak’s multiple comparison test, mean ± SEM, ** *p* < 0.01, (**D**) Neurite length analysis of DRG cultured after 20% 0.5 Hz stretch of CNS-nerve explant compared to static control. Stretch appeared to have a negative impact on the neurite outgrowth of these cells as well, although not statistically significant due to high variability. Paired two-tailed Student’s *t*-test, mean ± SEM of paired independent experiments indicated by the colors. (**E**) Diameter analysis showing a reduction trend in the outgrowth of all DRG populations after 20% stretch of the CNS-nerve explant. Two-way ANOVA with post-hoc Sidak’s multiple comparison test, mean ± SEM, ** *p* < 0.01.

Importantly, it was found that the 20% mechanical stretch of the DRG-nerve explant did not have a beneficial regenerative effect on the cultured DRG as the 10% mechanical stretch did on either PNS or CNS stretched DRG. Notably, 20% stretch of PNS-DRG explants had a statistically significant reduction of the axonal outgrowth of large-diameter neurons (Figure 4).

### 3.3. Unlike 20% Stretch, 10% Stretch Does Not Drastically Change the Cytoarchitecture of the Subjected Nerves

**Figure 5:**
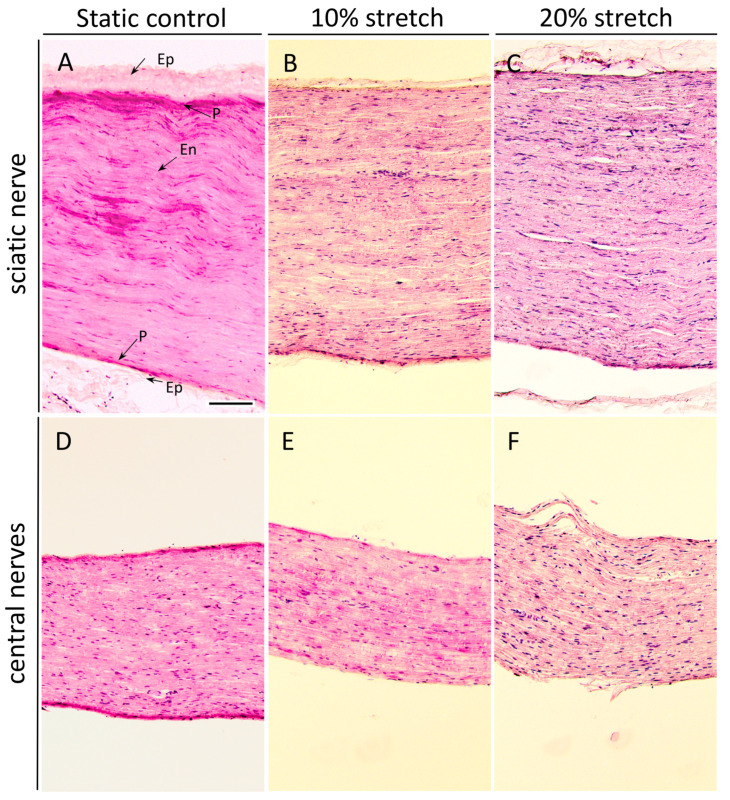
3 h of 10% stretch does not significantly alter the microarchitecture of the nerve, although 20% stretch does. (**A**–**F**) Photomicrographs (10×) of H&E staining of sciatic nerves (**A**–**C**) and central nerves (**D**–**F**). Nerves were fixed and dissected longitudinally after either no stretch (**A**,**D**) or mechanical loading of 10% (**B**,**E**) or 20% (**C**,**F**). Arrows in (**A**) indicate the epineurium (Ep), perineurium (P), and endoneurium (En). The epineurium, specific to the PNS, is densely formed in the static controls but begins to unravel upon 20% stretch. The perineurium, a protective sheath covering nerve fascicles, was found to slowly thin with increasing amounts of stretch, with some tears in the 20% samples. Notably, the naturally occurring nerve undulation observed in the static control (Bands of Fontana) is progressively lost upon the mechanical stretch. Scale bar, 500 μm.

Nerve sections were analyzed histochemically with H&E staining to examine the direct result of our treatment on the cytoarchitecture of the nerves. The cytoarchitecture of the nerves consists of the outermost layer of connective tissue, called epineurium, surrounding the inner layers of the perineurium and the endoneurium, the extracellular matrix and connective tissues surrounding and protecting the nerve fascicles and the individual nerve fibers, respectively. Specific characteristics of the morphology of the sciatic nerve are the undulating course of the nerves and connective tissue in the endoneurium. This results in a wavy structure, described first by F. Fontana in 1781 [24], observed only in peripheral [25] and not central nerves [26,27]. Interestingly, we found that 3 h of mechanical loading with an amplitude of 10% of the sciatic nerves or the central nerves did not have a drastic impact on any of the protective nerve layers (Figure 5B,E), but 20% stretch caused the unraveling of the epineurium in the sciatic nerves and thinning of the perineurium and more dramatically in the central nerves tear damage of the endoneurium was observed (Figure 5C,F).

### 3.4. 10%, but Not 20%, Stretch Enhances Neurite Outgrowth of Mechanoreceptors and/or Proprioceptors but Not Nociceptors

**Figure 6:**
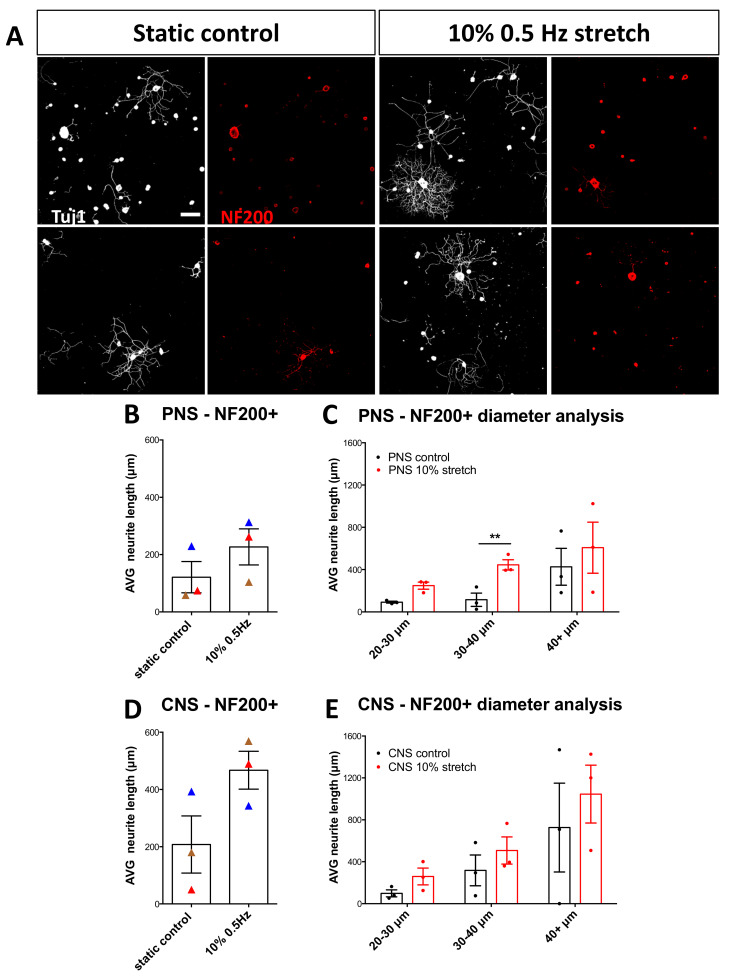
NF200+ DRG neurons respond to mechanical stretch. (**A**) Immunocytochemical analysis of DRG stained for NF200 after either no stretch or mechanical stretch (10% 0.5 Hz). Scale bar, 200 μm (**B**,**D**) Neurite length analysis of NF200+ PNS (**B**) and CNS (**D**) DRG upon 10% 0.5 Hz mechanical stretch. Paired two-tail Student *t*-test, mean ± SEM of paired independent experiments indicated by the colors. (**C**,**E**) Diameter analysis of PNS (**C**) and CNS (**E**) DRG outgrowth comparing the static controls to the stretched explants shows that after peripheral stretch 30–40 µm-diameter neurons increase their neurite outgrowth. Two-way ANOVA with post-hoc Sidak’s multiple comparison test, mean ± SEM, ** *p* < 0.01.

Given that mechanical stretch seems to affect the outgrowth of larger-diameter neurons in vitro in particular, we sought to validate the DRG subpopulation most susceptible to such treatment. Therefore, we began staining with neurofilament 200 (NF200), which stains all A- myelinated fibers [28,29]. Although we observed an overall trend, significance was not reached, which may be due to the high variability with this staining that contains medium Aγ peptidergic nociceptors and medium to large Aβ mechanoreceptors as well as large Aα proprioceptors (Figure 6A,B). Interestingly, when diameter analysis was performed, the 30–40 µm group of the PNS nerve-DRG explant did respond with increased neurite outgrowth. It should be noted that explant and primary culture conditions coupled with the lower cell numbers in the medium to large diameter cell populations leads to high variability between biological replicates.

**Figure 7:**
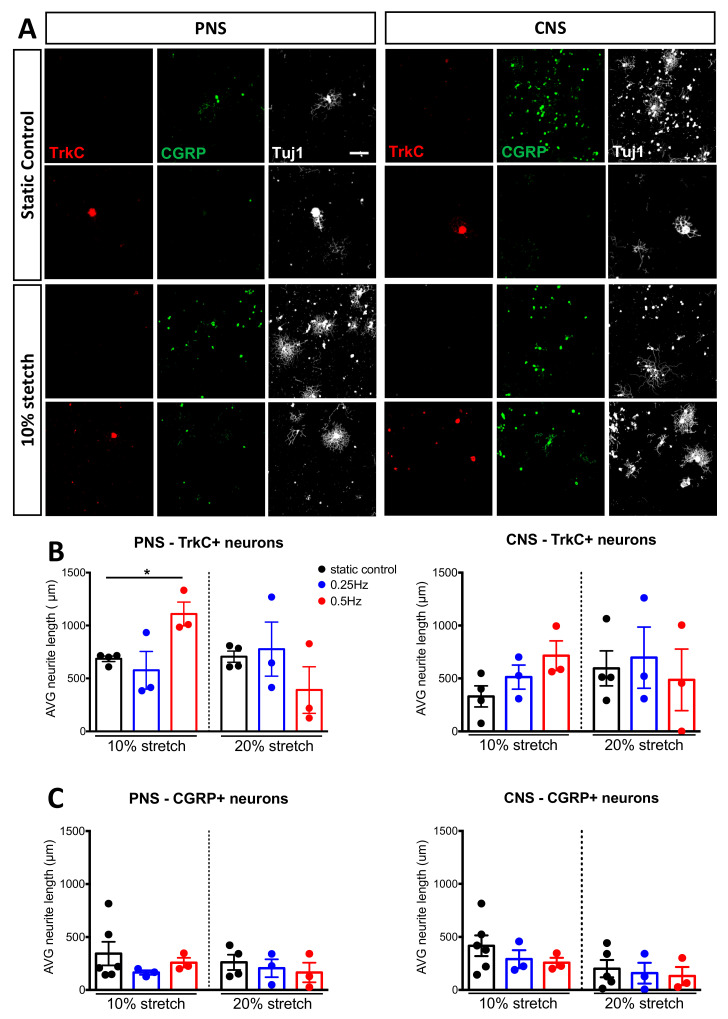
TrkC+ but not CGRP+ DRG neurons respond after mechanical stretch. (**A**) Immunocytochemical analysis of DRG stained either for TrkC (green) or CGRP (red) after either no stretch or mechanical stretch (10% 0.5 Hz). Scale bar, 200 μm (**B**) Neurite length analysis of TrkC+ DRG upon mechanical stretch with an amplitude of either 10% or 20% and a frequency of 0.25 Hz or 0.5 Hz. (**C**) Neurite length analysis of CGRP+ DRG upon mechanical stretch with an amplitude of either 10% or 20% and a frequency of 0.25 Hz or 0.5 Hz. Two-way ANOVA, with post-hoc Sidak’s multiple comparison test, mean ± SEM, * *p* < 0.05.

To differentiate the various populations further, we turned to the antibodies for TrkB (Tropomyosin receptor kinase B, the receptor for brain-derived neurotrophic factor and neurotrophin-4 and a marker for mechanoreceptive neurons), parvalbumin (a calcium-binding albumin protein and marker for proprioceptive neurons) and Calcitonin gene-related peptide (CGRP, a marker of peptidergic nociceptive neurons) [30]. Unlike our previous experience with TrkB in tissue sections, in vitro, we struggled to observe specific staining. Parvalbumin failed to stain enough cells for proper quantification. For this reason, we stained large-diameter proprioceptive and mechanoreceptive neurons [31] with TrkC, which is the neurotrophin-3 receptor [32]. We observed that TrkC+ neurons measured on average between 49–57 μm in all experiments conducted. Moreover, we found that TrkC+ neurons did grow after peripheral nerve stretch (Figure 7A,B). This observation was only with 10% at 0.5 Hz, not with lower frequency (0.25 Hz) or increased stretch percentage (20%).

Previously, hindlimb stretching of spinal cord injured rats led to increased sprouting of nociceptive fibers in the dorsal horn of the spinal cord [33]. Therefore, in addition to differentiating Aδ peptidergic nociceptive fibers from the other A-fibers, we investigated if mechanical stretch could enhance the outgrowth of nociceptive neurons. For this, we stained DRG following stretch or static conditions with CGRP (Figure 7A). On average, the CGRP+ neurons measured 26–27 μm between different experiments. Under our standard amplitude (10%) conditions, axonal outgrowth of CGRP+ neurons were not altered from either PNS or CNS stretch at either frequency examined (Figure 6C). As extreme stretching of nerves is known to induce pain, we examined if a higher amplitude (20%) of stretch would induce the outgrowth of nociceptive neurons [34]. Figure 7C shows again no changes were observed. Therefore, we can eliminate nociceptive neurons in response to mechanical stimulation and conclude our approach of 10% 0.5 Hz mechanical stretch affects larger-diameter neurons, including proprioceptive and mechanosensitive neurons.

### 3.5. ATF3 Is Increased by 3 h 10% Mechanical Loading

**Figure 8:**
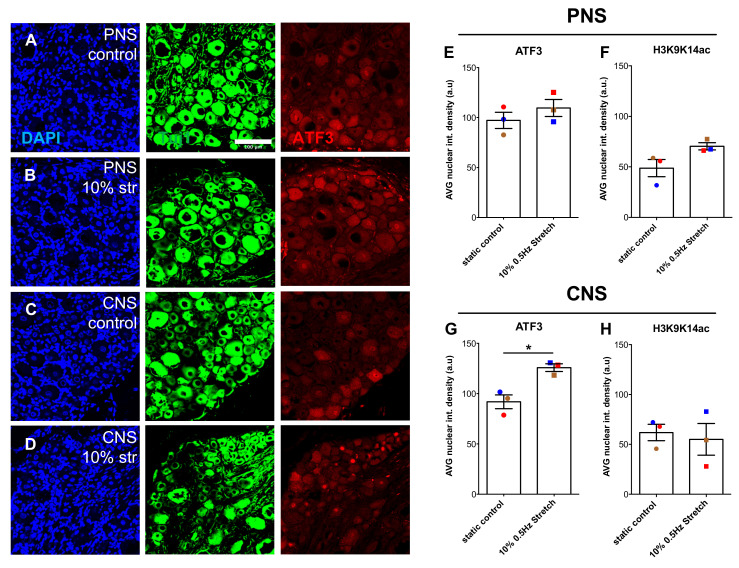
Within 3 h of 10% mechanical stretch, ATF3 nuclear DRG levels increased in the CNS DRG-nerve explant. (**A**–**D**) Representative immunofluorescence photomicrographs (20×) of DRG sectioned either 3 h post- no stimulation (**A**) or 10% mechanical stretch (**B**) of PNS DRG-nerve explants and no stimulation (**C**) or 10% mechanical stretch (**D**) of CNS DRG-nerve explants. DRG were stained for DAPI (blue), βIII-tubulin (Tuj1) (green), and ATF3 (red). Scale bar, 100 μm. (**E**,**G**) Nuclear intensity density analysis of ATF3 in DRG derived from PNS DRG-nerve explants (**E**) and CNS DRG-nerve explants (**G**). Paired two-tailed Student *t*-test, mean ± SEM of paired independent experiments indicated by the colors, * *p* < 0.05. (**F**,**H**) Nuclear intensity density analysis of H3K9K14ac in DRG derived from PNS DRG-nerve explants (**F**) and CNS DRG-nerve explants (**H**). Paired two-tailed Student *t*-test, mean ± SEM of paired independent experiments indicated by the colors.

Thus far, we have shown that 10% cyclic mechanical stretch of both PNS- and CNS-nerve explants managed to increase the axonal outgrowth of cultured larger diameter mechanoreceptive and proprioceptive neurons without a dramatic impact on the cytoarchitecture of the subjected nerves. However, the underlying mechanisms involved in the mechanical stimulation of axonal outgrowth remain unknown. A peripheral nerve injury induces a regenerative signal that promotes long-distance regeneration of injured neurons. A plethora of factors are involved in this signal leading to post-translational modifications of cytoplasmic proteins [35] as well as epigenetic and transcription factor changes leading to expression of regeneration-associated genes [36,37,38]. An integral factor in this regenerative signal is Activating Transcription Factor 3 (ATF3) [39,40], whose nuclear levels increase after a peripheral injury and contribute to the regeneration of injured peripheral neurons [41]. In other non-neuronal models of cyclic stretch, ATF3 was found to be increased early on by gene expression and nuclear protein translocation as well as found to be integral to the beneficial effects that followed [42]. Modification of histones is an initiation signal of regenerative gene expression changes [43,44,45] and can be activated in animals that participate in voluntary wheel running in an enriched environment [46]. Therefore, we investigated whether 3 h 10% 0.5 Hz mechanical stretch immediately affects ATF3 regulation or histone 3 lysine 9 lysine 14 acetylation (H3K9K14ac) by DRG section immunohistochemistry. Remarkably, even in the short 3-h 10% stretch period of CNS DRG-nerve explants, significant enhancement of ATF3 nuclear levels was observed (Figure 8A,B,E,G). Further diameter analysis did indicate significance by Two-Way ANOVA for stretch treatment and diameters, but not by post-hoc multiple comparison for specific subpopulation enhancement. Although a trend appeared, no significant changes were seen with H3K9K14ac (Figure 8C,D,F,H). Further, there was no significance when Two-way ANOVA examined diameter analysis for H3K9K14ac.

## 4. Discussion

Mechanical forces upon and within developing neurons determine towing neurite outgrowth [47] axonal pathfinding [48] and ultimately “stretch growth” of integrated axonal tracts [11]. Moreover, muscle contractions can guide sensory axons in zebrafish [49]. Although all neurons are constantly under tension in vivo, in the periphery this tension is increased by limb movement [50]. In fact, cyclic stretch produced by exercise is a common rehabilitative approach following a neuronal injury in the PNS and the CNS [51,52]. Interestingly, it has been demonstrated that treadmill training can prime dissected DRG to enhance axonal outgrowth ex vivo and increase regeneration of sciatic nerves upon crush [53]. Similarly, voluntary wheel running within an enriched environment led to proprioceptive growth [46]. Passive exercise and manual stimulation provide similar effects to exercise training [54,55,56]. Physical exercise contributes to the rate of axonal growth as well as the number of growing axons [57]. An underlying reason why exercise-trained injured animals have less misdirection of regenerating axons [57] could be that often neurite growth is found in the direction of applied stretch [58].

The beneficial effect of exercise is not limited to the recovery of the PNS, rhythmic exercise also leads to sensory [59] and motor [60] functional recovery following spinal cord injury, and is associated with upregulated levels of known neurotrophins and enhanced sprouting [59,61]. Interestingly, Sachdeva and colleagues showed that cyclic exercise combined with peripheral grafts leads to the upregulation of RAGs (GAP-43, β-actin, and neuritin) and enhanced regeneration of propriospinal, but not sensory neurons [62]. It should be noted this is likely due to non-cell-autonomous mechanisms. Remarkably, when the flexibility of the vertebral column is increased as that of the lamprey (along with lack of myelin sheathing), spinal cord regeneration is observed even after multiple injuries [63], and further enhanced by swim training [64]. Swimming led to an increase of 5% strain on the spinal cord from non-stress situations [65]. Moreover, small caliber reticulospinal and propriospinal axons were the first to regrow before larger reticulospinal axons [66]. It can be postulated that smaller caliber axons may be more receptive to mechanical strain. For example, the injury-inducing mechanical strain applied on DRG showed that CGRP+ neurons, which represent the majority of the thin unmyelinated C-fibers [28,67], show higher levels of Caspase-3 activation and increased levels of Caspase-3-dependent apoptosis compared to NF200+ myelinated neurons [68], which represent larger diameter A-fibers [28]. Notably, other neurons residing in the CNS also show the capacity to respond to a mechanical tension. Retinal ganglion cells (RGCs) do not regenerate spontaneously following optic nerve crush and most of them undergo apoptosis [69]. Interestingly, it has been shown that these cells can respond to mechanical towing by elongating their axons [70] coupled to the release of ATP and cytokines acting in an autocrine manner to enhance their survival [71,72]. Although these results derive from cultures of dissociated RGCs, it is clear that mechanical tension can have an impact on the survival and growth of CNS neurons. Therefore, it would be of interest to adopt our stretch parameters and setup to the optic nerve, although the length of the rat optic nerve may pose some technical difficulties.

Here, for the first time, we used DRG explants attached either to their peripheral or their central branches applied with uniaxial sinusoidal mechanical stretch, imitating the cyclic nature of a limb movement and the corresponding physiological forces. Stretch parameters could be kept consistent and this allowed us to compare the responsiveness of sensory adult neurons to the same levels of mechano-stimulation of their peripheral or central branches. Our results showed for the first time that 10% stretch can promote axonal outgrowth of both PNS and CNS large-diameter DRG neurons. The overall effect was clear in centrally stretched DRG neurons, but further analysis showed that NF200+ and TrkC+ peripherally stretched DRG neurons also showed significant differences. Similar to what was observed with exercise training, we found that cyclic stretch of the CNS nerve-DRG explant not only increased neurite outgrowth but also enhanced initiation of neurite outgrowth. Notably, when we applied higher amplitude stretch (20%) this did not lead to any higher regenerative effect, but instead, it damaged the subjected nerves similar to that of high-intensity training [57]. Possibly an intermediate level of stretch at 12% or 15% would be worth exploring further.

Physiologically, peripheral nerves may be built to resist stretch forces since they are not protected by bony structures. Interestingly, some peripheral nerves are more resistant to mechanical stretch than others [73]; with one example of peripheral nerves in the tongue of whales showing extreme elastic properties [74]. In general, it seems that peripheral nerves have been evolved to withstand forces due to their anatomy [74] as, for example, central nerves do not form fascicles held together by connective tissue forming the epineurium. However, regardless of where the nerves are located, either in the PNS connected to muscle/skin or CNS behind a rigid bony structure, we observed they retain their capacity to respond to mechanical input. Although there are differences in the anatomy of the PNS and CNS nerves, it did not appear to hinder the response to stretch but may have contributed to higher levels of variability between experiments. In contrast to exercise regimes, the sciatic nerve disconnected from muscles and skin was able to sense mechanical tension. Central nerves, not directly connected to either muscle or skin, also responded to mechanical tension. Although this does not prove cell autonomy in response to stretch, it does narrow the possibilities to the cellular composition of the nerves themselves.

This response is limited to a subset of DRG neurons that normally code mechanical stimuli, as mechanoreceptors respond to mechanical stimuli and proprioceptors to muscular and visceral pressure. It should be noted again that these cells represent the minority of the DRG sensory neuronal pool, and this contributes to the high variability observed in results regarding these populations. Unlike previous work with exercise training, our work is unique in that we attempted to examine which population/s of sensory neurons responded to mechanical tension. After peripheral injury, neurochemical markers were found to change in the DRG [28]. It is possible in cell culture that neurochemical markers either do not work or change from standard in vivo conditions. It should be noted the distribution of these markers are wide in diameter analysis [75] and make it difficult for conclusive subpopulation predictions. Either dual markers to further ensure the classification of neurons or single-cell transcriptomic analysis of DRG following mechanical stretch is required. This would additionally allow for a better understanding of the mechanisms involved in stretch-associated regenerative pathways of each DRG subpopulation. Given that disassociation of the cells for culture did not alter the stretch-induced mechanisms, this approach should be feasible for this type of experimentation.

Although classically known as a stress-related protein, it has been shown that ATF3 mRNA levels are upregulated 6 h upon a regenerative injury [40]. Additionally, ATF3 protein levels increase after treadmill training in rats enhancing axonal regeneration of the injured sciatic nerves [76]. We have shown that 3 h of ex vivo mechanical axonal stretch was sufficient for significantly increasing nuclear localization of ATF3 in centrally stretched DRG compared to non-stretched controls. Previously, upstream MAPK pathways were found to induce ATF3 which in turn may lead to GAP-43 expression in exercise paradigms [76]. In other cell models, cyclic stretch is known to activate mechano-signaling (channel activation and Ca^2+^ influx) within a minute, then signaling cascade activation over several minutes (ROS production and MAPK signaling), followed by increased transcription factor activation from minutes to the first hour after stretch has begun, and cytoskeletal remodeling follows up to 6 h [77]. It would be of interest to understand if the same pathways play a role in our nerve stretch paradigm as well. To further test the requirement of de novo transcription to the regenerative stretch effect, we could use transcriptional inhibitors, such as actinomycin-D, during the culturing state.

It would be interesting to investigate the regulation of the extracellular matrix (ECM) upon mechanical loading of neurons. Recent studies by Nichols and Smith have shown axotomized DRG in zebrafish can reenter the spinal cord when F-actin-based invasion components are stabilized through the activation of Src and the release of matrix metalloproteases (MMPs) [78,79]. Although shown in endothelial cells, mechanical stretch can activate Src [80,81], thus it would be interesting to investigate whether cyclic mechanical loading can activate this pathway in stretched DRG. MMPs are known to regulate the regeneration of neuronal cells by remodeling the ECM [82,83,84,85]. Cyclic stretch (17–18%, 0.5Hz) of lung microvascular endothelial cells resulted in elevated levels of MMP-1 and MMP-2 [86]; stretching (15%) of retinal glial (Müller) cells led to elevated levels of MMP-2 [87] but their connection to neuronal mechanical stretch is poorly reviewed. Based upon our results and the fact that neurons are mechanoresponsive cells it would be interesting to investigate the expression of MMPs upon DRG explant mechanical stretch and their contribution to the enhanced outgrowth of large-diameter DRG neurons.

Exercise-induced axonal growth requires the upregulation of neurotrophins, such as BDNF [88], NT-4/5 [89], NT-3 [53], produced by neurons, muscles, and Schwann cells. Contrastingly, paralysis leads to decreased BDNF levels [61]. As exercise-induced neurotrophin signaling was non-cell-autonomous to growing axons in the CNS [62], it would be of interest to examine neurotrophin signaling in our axonal stretch model to elucidate if they are specifically responsive to axonal stretch alone or other components of exercise.

Through mechanotransduction, cells translate mechanical forces into biological responses [90]. The neuronal cytoskeleton plays a role in this process, linked to integrin-based focal adhesions [90]. Integrins have been shown to be important to the neuroregenerative process in the PNS and are now also induced to allow regeneration of the CNS [91]. Further understanding the role of integrin biology in our model may allow us to unlock the mechanism underlying axonal stretch growth. For example, calcium influx from stretch-gated ion channels, such as transient receptor potential canonical 1 (TRPC1), found in the PNS and CNS, can lead to activated cleavage of talin, which links actin to integrins, important for spinal axonal growth [92]. Further, the TRP, subfamily V, member 2 (TRPV2) channel is also activated by stretch and is expressed in motor and sensory neurons during development regulating their outgrowth [93]. In adult animals, TRPV2, also known as vanilloid-receptor-like protein 1 (VRL-1), is expressed in Aδ- or Aβ-fibers in the DRG [93]. Therefore, it would be useful to examine if blockade of such channels could abolish the stretch regenerative effect and if differences in the density of such channels on the axons of central and peripheral nerves could explain their responsiveness to mechanical stimulation.

This proof-of-concept work was initiated to specifically address if cyclic stretch is directly applied to the nerve, particularly in nerves lacking this input, would this lead to enhanced neurite outgrowth. We have proven that it does and in a specific subpopulation of cells (mechano- and proprioceptors) with 10% stretch 0.5 Hz, but not greater amplitudes or lower frequencies. Although we identified a mechanism underlying axonal cyclic stretch growth (ATF3), in the future a better understanding of axonal stretch growth pathways could lead to new avenues of intervention in neuroregenerative science.

## Figures and Tables

**Table 1:** Stretch parameters examined.

% Stretch, Frequency (Hz)	Time of Stretch (h)	Recovery Time (h)
2.5%, 0.5 Hz	3 h	0 h
5%, 0.5 Hz	6 h	0 h
5%, 0.5 Hz	3 h	3 h
5%, 0.5 Hz	3 h	0 h
10%, 0.25 Hz	3 h	0 h
10%, 0.5 Hz	3 h	0 h
20%, 0.25 Hz	3 h	0 h
20%, 0.5 Hz	3 h	0 h

## Supplementary Materials

The following are available online at https://www.mdpi.com/2073-4409/10/1/32/s1, File S1: Galiltools code used for the control of the oscillator/bioreactor movement. The code refers to 1 mm dislocation (10% stretch) with 0.5 Hz frequency.
