# Supplementary material for: Cyclic Stretch of Either PNS or CNS Located Nerves Can Stimulate Neurite Outgrowth
Source: Cells. 2020 Dec 28;10(1):32. doi: 10.3390/cells10010032 (PMC7824691; doi:10.3390/cells10010032)
Supplement: Supplementary file 1 [file cells-10-00032-s001.pdf]

Galiltools code used for the control of the oschilator/bioreactor movement. The code below refers to 1 mm dislocation (10% stretch) with 0.5Hz frequency.

```
#OSZI01
WT2000
NO______________REFERENCE MOVE____________________________________________
NO
SHX
DP0;NO              SET POSITION COUNTER TO ZERO
#INDEX
NO                  MOVE TO FIND HARD END
JG20000
BGX
MG"SUCHE HARDANSCHLAG"
#LOOP1;NO           CHECK BUILDUP OF POSITION ERROR
JP#LOOP1,_TEX<10000;NO    IF NO MORE MOOVEMENT EXIT LOOP1
NO
STX
AMX
NO                  FIND INDEX
JG-10000
MG"STARTE INDEX SUCHLAUF"
FIX
BGX
AMX
MG"INDEX GEFUNDEN, NULLPUNKT GESETZT"
WT20000;NO          WAIT 20 SEC
NO
NO______________SINUSOIDIAL MOVE PROGRAMM________________________________
NO
#SINUS
NO                  SETTING VECTOR MOVE PROPERTIES
VM XN;NO            N IS THE VIRTUAL AXIS
```

```
VA 68000000
VD 68000000
VS 31416;NO                    VS=2*PI*RADIUS*f

NO                          DOING 10 CYCLES (3600 DEG)
#KREIS
CR 10000,90,3600;NO           RADIUS, START DEG., STOP DEG.
VE
BGS
AM
JP#KREIS;NO                   INFINITE LOOP
EN
NO_____________PROGRAMM END___________________________________________
```
